# The Effects of Deoxynivalenol and Zearalenone on the Pig Large Intestine. A Light and Electron Microscopy Study

**DOI:** 10.3390/toxins10040148

**Published:** 2018-04-04

**Authors:** Barbara Przybylska-Gornowicz, Bogdan Lewczuk, Magdalena Prusik, Maria Hanuszewska, Marcela Petrusewicz-Kosińska, Magdalena Gajęcka, Łukasz Zielonka, Maciej Gajęcki

**Affiliations:** 1 Department of Histology and Embryology, Faculty of Veterinary Medicine, University of Warmia and Mazury in Olsztyn, Oczapowskiego 13, 10-719 Olsztyn, Poland; lewczukb@uwm.edu.pl (B.L.); mprusik@gmail.com (M.P.); marysia-h@wp.pl (M.H.); marcelapetrusewicz@wp.pl (M.P.-K.); 2 Department of Veterinary Prevention and Feed Hygiene, Faculty of Veterinary Medicine, University of Warmia and Mazury in Olsztyn, Oczapowskiego 13, 10-719 Olsztyn, Poland; mgaja@uwm.edu.pl (M.G.); lukaszz@uwm.edu.pl (Ł.Z.); gajecki@uwm.edu.pl (M.G.)

**Keywords:** mycotoxins, zearalenone, deoxynivalenol, histology, ultrastructure, large intestine, pig

## Abstract

The contamination of feed with mycotoxins results in reduced growth, feed refusal, immunosuppression, and health problems. Deoxynivalenol (DON) and zearalenone (ZEN) are among the most important mycotoxins. The aim of the study was to examine the effects of low doses of these mycotoxins on the histological structure and ultrastructure of the large intestine in the pig. The study was performed on 36 immature gilts of mixed breed (White Polish Big × Polish White Earhanging), which were divided into four groups administrated per os with ZEN at 40 µg/kg BW, DON at 12 µg/kg BW, a mixture of ZEN (40 µg/kg BW) and DON (12 µg/kg BW) or a placebo. The pigs were killed by intravenous overdose of pentobarbital after one, three, and six weeks of treatment. The cecum, ascending and descending colon samples were prepared for light and electron microscopy. Administration of toxins did not influence the architecture of the mucosa and submucosa in the large intestine. ZEN and ZEN + DON significantly decreased the number of goblet cells in the cecum and descending colon. The mycotoxins changed the number of lymphocytes and plasma cells in the large intestine, which usually increased in number. However, this effect differed between the intestine segments and toxins. Mycotoxins induced some changes in the ultrastructure of the mucosal epithelium. They did not affect the expression of proliferative cell nuclear antigen and the intestinal barrier permeability. The obtained results indicate that mycotoxins especially ZEN may influence the defense mechanisms of the large intestine.

## 1. Introduction

Zearalenone (ZEN) and deoxynivalenol (DON) are mycotoxins produced by *Fusarium* fungi. Their wide distribution in nature and negative effects of consumption of food and feed contaminated with ZEN and DON for human and animal health mean that they have become a subject of extensive research [1]. The toxic outcome of these mycotoxins includes feed refusal, reduced growth, gastrointestinal lesions, immunosuppression, and reproductive disorders [2]. At the cellular level, ZEN and DON can negatively affect numerous pathways and processes. Many aspects of mycotoxins impacting on humans and animals especially in the case of mixed mycotoxicosis are still not fully recognized. Animal species show differential sensitivity to mycotoxins.

Deoxynivalenol (DON, IUPAC name: (3α,7α)-3,7,15-trihydroxy-12,13-epoxytrichothec-9-en-8-one) belongs to type B trichothecene mycotoxins and is the most commonly detected toxin of this group. DON in acute and moderate to low doses induces toxic and immuno-toxic effects in a variety of cell systems and animal species [3]. The differential susceptibility to DON in animal species evaluated to date presumably depends on differences in metabolism, absorption, distribution, and elimination of DON among animal species [4]. Pig is the most sensitive livestock species to DON. Absorption of DON in pigs is rapid and the toxin reaches peak plasma concentrations within 30 min of oral administration [5]. Results of Dänicke et al. [6] showed that, in pigs, a majority of the ingested DON is absorbed in the proximal part of the small intestine. In the large intestine, de-epoxidation takes place, which does not contribute much to detoxification. DON is primarily excreted in urine in free and conjugated forms and much smaller amounts of the toxin are excreted in feces as de-epoxidase and free forms [6,7]. Research provides evidence that the chronic ingestion of DON, also in low doses, alters the small intestine morphology affecting the mucosal epithelial cells and the villi [8,9,10]. DON modulates the immune responsiveness of the intestinal mucosa, the cytokine production, and the cross-talk between epithelial cells and the intestinal immune cells [11]. DON reduces the expression of the adherent junction protein and the tight junction protein in the small intestine [12]. In the large intestine, DON in a dosage of 1008 µg/kg of feed modified the local immune response by changing the expression of toll-like receptors [13].

Zearalenone (ZEN, IUPAC name: (3S,11E)-14,16-dihydroxy-3-methyl-3,4,5,6,9,10-hexahydro-1H-2-benzoxacyclotetradecine-1,7(8H)-dione) and its metabolites have estrogenic activity and compete with endogenous hormones for the binding sites of estrogen receptors [14]. ZEN also influences the activities of enzymes involved in steroid metabolism: 3-β-hydroxysteroid dehydrogenase type 1, cytochrome P450 side-chain cleavage enzyme, and P450 aromatase. Treatment with ZEN leads to precocious puberty, reproductive disorders, and hyperestrogenizm [15]. ZEN and its derivatives impair the inflammatory response and, therefore, may affect the capacity of organisms to both eradicate injurious stimuli and initiate the healing process [16]. The small intestine absorbs ZEN first so it is exposed to a high concentration of the toxin, which has the deleterious effect on its morphology even in the case of low doses [17,18]. In pig, the total biological recovery of ZEN in feces is about 34% ± 3% [19] and taking into account the slow passage of digestion in the large intestine ZEN can influence the structure and function of this part of the gastrointestinal tract as well. However, this aspect of ZEN intoxication has not been investigated. 

The toxicity of mycotoxins needs to be addressed in the context of mycotoxins mixtures to assess health risks [20], however the effects of mycotoxin combination are poorly known. Existing data indicate that the type of interaction depends on the type of toxins, the ratio between toxins, and the concentration of the toxin mixture at a constant ratio [20,21].

In many cases, a diet contains DON and ZEN together [22]. However, the effects of concurrent exposition to these mycotoxins are still poorly recognized and difficult to predict [8,23]. So far, both additive and non-additive effects were found when DON and ZEN were administrated together [24]. DON + ZEN in combination were toxic on swine jejunal epithelial cells at concentrations nontoxic when administrated individually [20,24]. On the other hand, no additive effects of these toxins on the height of villi and the percentage of goblet cells in crypts of the pig jejunum were observed [18].

The intestinal tract is the main barrier to ingested feed contaminants. The intestine possesses three basic protective mechanisms—physical, chemical, and immunological. Several reports have demonstrated that mycotoxins are able to compromise these mechanisms. Most of the studies concern the small intestine, but very little is known about the effect of mycotoxins on the large intestine. On the other hand, the presence of DON and ZEN derivatives and non-metabolized mycotoxins in digesta of the large intestine along with the slow passage of content through this part of the gastrointestinal tract result in a long-time of mucosa exposure to the toxins [25]. 

The aim of the present study was to determine the effects of DON (12 µg/kg BW) and ZEN (40 µg/kg BW) when fed, individually and in combination, to the pigs for one, three, or six weeks on the cecum and colon histology and ultrastructure. Moreover, the effect of mycotoxins on the permeability of the intestinal epithelial barrier was studied using the lanthanum method. Additionally, immunocytochemical study of proliferative cell nuclear antigen (PCNA) was performed to assess the effect of toxins on the proliferation of the intestinal epithelial cell.

The selection of doses used in our experiment was previously widely discussed [17,18,26]. Briefly, the dosage of DON used in our study, 12 µg/kg BW, was much lower than No Observed Adverse Effect Level (NOAEL) proposed for pigs [27]. The dose of ZEN used in this study, 40 µg/kg BW, was the same as the NOAEL established by—The joint FAO/WHO Expert Committee on Food Additives for pigs [28]. 

## 2. Results and Discussion

### 2.1. Light Microscopy Study

#### 2.1.1. Architecture of the Mucosa

The cecum, colon ascending and descending of the control pigs were characterized by numerous, well-developed mucosal crypts that span the depth of the lamina propria. The columnar epithelium lining the crypts and covering the luminal surface comprised primarily goblet cells and absorptive cells. The lamina propria consisted of the stromal elements and cells of the immune system. The well-developed muscularis mucosa separated the mucosa from the tunica submucosa, which had loose arrangement and contained numerous adipocytes. The muscularis and the serosa were typical for these segments of the large intestine in pigs. No noticeable qualitative differences were observed in the architecture of the cecum and both colon regions between the control group and the groups of pigs treated with mycotoxins. 

**Figure 1:**
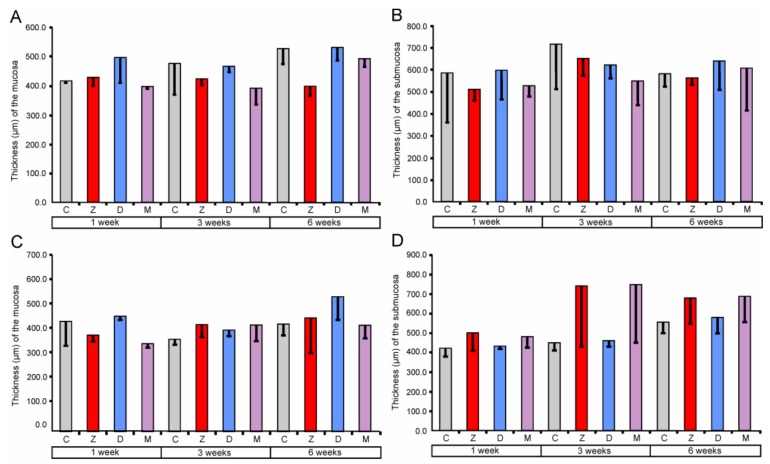

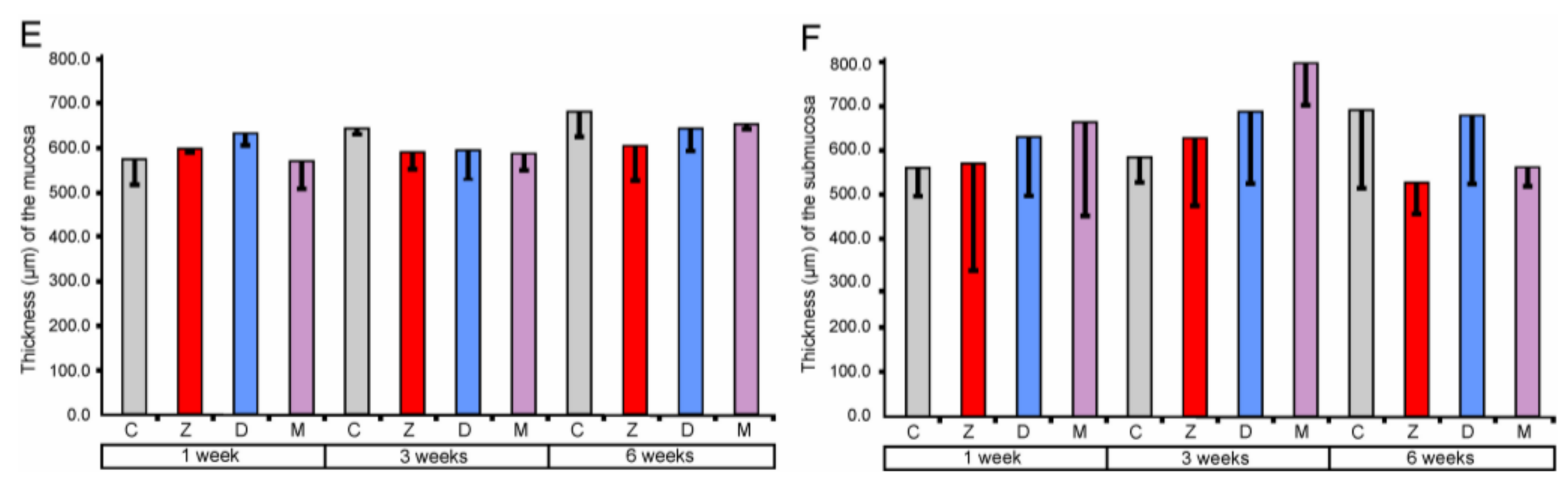
Morphometric characteristic of the mucosa and submucosa of the large intestine segments. Thickness of the cecum mucosa (**A**) and submucosa (**B**), ascending colon mucosa (**C**) and submucosa (**D**), and descending colon mucosa (**E**) and submucosa (**F**). Values presented are means and the standard errors of the mean (SEM). The capital letters under horizontal axis, C—control group, Z—group treated with zearalenone (ZEN), D—group treated with deoxynivalenol (DON), and M—group treated with ZEN + DON.

Morphometric analysis demonstrated no significant differences in the thickness of mucosa and submucosa between the investigated groups of pigs (see Figure 1).

The obtained data showed that ZEN and DON as well as ZEN + DON had no effect on the qualitative and quantitative characteristics of the mucosa and submucosa in all regions of the large intestine investigated. Previous studies indicated that the dosages of mycotoxin used in our experiment affected histology of the small intestine. However, the significant quantitative changes were noted exclusively after six weeks of the treatment [17,18]. The thickness of the duodenum submucosa was significantly higher in the pigs receiving DON + ZEN for six weeks than in the control pigs [17]. In the jejunum, the thickness of the mucosa was affected in the groups treated by DON and ZEN + DON for six weeks [18]. So far, there has been no research regarding the effects of ZEN and DON on the large intestine architecture. 

#### 2.1.2. Goblet Cells

**Figure 2:**
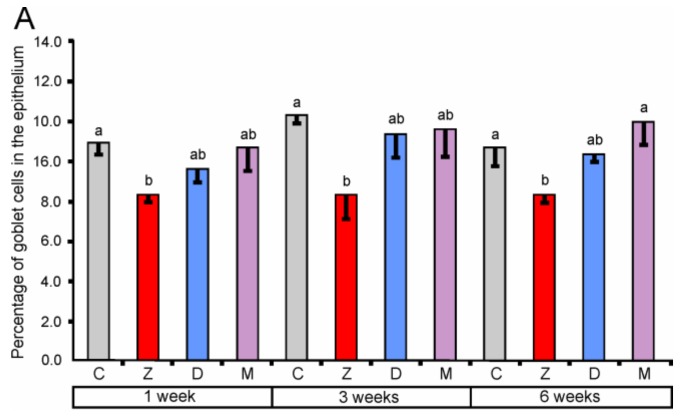

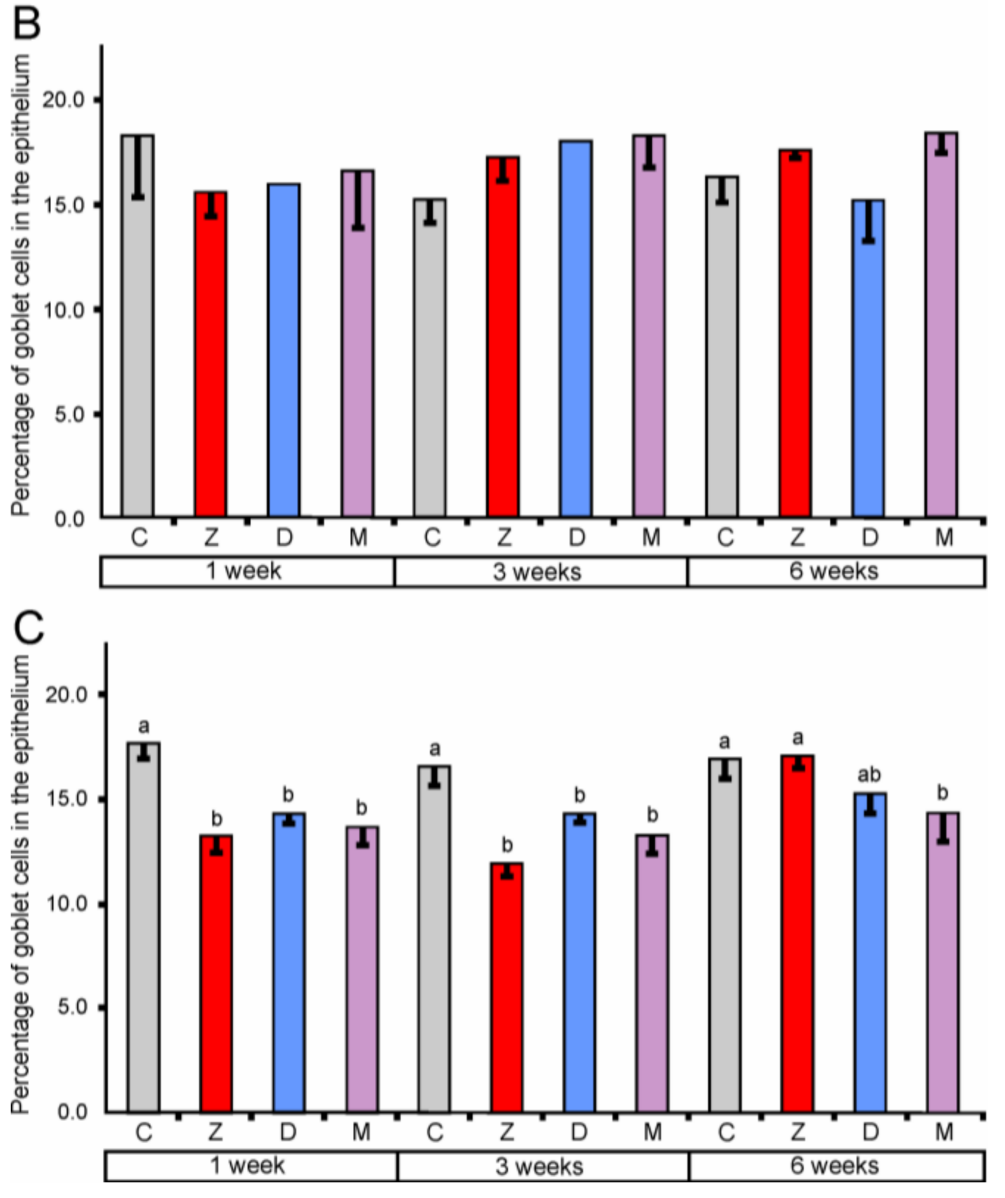
The percentage of goblet cells in the mucosal epithelium of the cecum (**A**), ascending colon (**B**), and descending colon (**C**). Values presented are means and SEM. Means annotated with different lower case letters above the bars are significantly different at *p* ≤ 0.05. For other explanations, see Figure 1.

The percentage of goblet cells after ZEN treatment for one, three, and six weeks in the cecum along with one and three weeks in the descending colon were significantly decreased (see Figure 2). The effect of DON was significant only in the descending colon after one and three weeks of treatment. Administration of ZEN + DON also resulted in a decrease in the percentage of goblet cells. The statistically significant effects were observed in the descending colon after one, three, and six weeks of the experiment.

Our results demonstrate that the mycotoxins used in the experiment especially ZEN decrease the number of goblet cells in the large intestine. It should be noted that mycotoxins have the most adverse influence on goblet cells in the descending colon. However, results indicate the complexity of this action and its dependence on many factors. 

Goblet cells are mucin-secreting cells forming the mucus layer that protects the mucosal surface. This layer is a result of the dynamic balance between the secretion of mucin by goblet cells and the degradation of mucin. Reduction in the goblet cell number is associated with disorders of intestinal protection [29,30]. The number of goblet cells changes under various factors [31]. Dietary components modulate the function of goblet cells and affect composition of mucin [30,31]. 

There are no data concerning the influence of mycotoxins on goblet cells in the epithelium of the large intestine. In the small intestine epithelium, the ingestion of the DON contaminated diets induced a significant decrease in the number of goblet cells in the pig jejunum and ileum [9,10,12]. On the other hand, more numerous goblet cells were found after administration of a diet containing ZEN [32]. Our previous results concerning the small intestine in the pigs treated with the same doses of ZEN and DON demonstrated that the effect of toxins is dependent on the part of the intestine and the duration of treatment [17,18]. No changes in goblet cells were found in the duodenum including the villus epithelium and the crypt epithelium [17]. However, in the jejunum, the percentage of goblet cells was transiently increased in the villus epithelium, but not in the crypt epithelium [18]. The differences between these two parts of the small intestine could be explained by the fact that large amounts of mucous are produced in the duodenum by Brunner’s glands. These glands showed hypertrophy after administration of DON + ZEN for six weeks [18]. It should be noted that the mucous system differs substantially between the small and large intestine and these differences concern both goblet cells and regulation of their secretion [33]. 

The decrease in the number of goblet cells observed in the present study suggests some discrepancy in mucous secretion and, as a consequence, the depletion in the protective mechanism in the large intestine was a result of mycotoxin treatment. The recent discoveries placed goblet cells at the center position of our understanding of mucosal biology and the immunology of the intestinal tract [33]. The mucous layer is stratified and organized as a filter that physically separates the bacteria from the epithelial cell [34]. Several studies lead to the conclusion that goblet cells form the major line of defense at the intestinal mucosa. However, this is far from a full understanding of the phenomenon [33,35,36].

#### 2.1.3. Lymphocytes and Plasma Cells

The effect of mycotoxins on the number of lymphocytes in the epithelium covering lumen and crypts of the large intestine, and in the lamina propria showed regional differences. 

**Figure 3:**
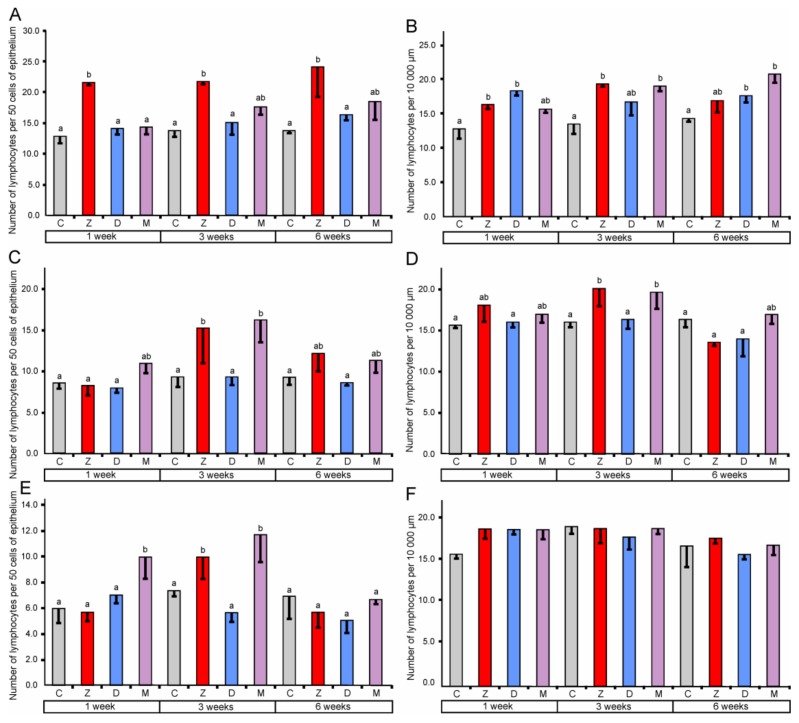
Number of lymphocytes in the mucosal epithelium (**A**) and in the lamina propria (**B**) of the cecum, in the mucosal epithelium (**C**) and in the lamina propria (**D**) of the ascending colon, in the mucosal epithelium (**E**) and in the lamina propria (**F**) of the descending colon. Number of lymphocytes in the mucosal epithelium was expressed per 50 epithelial cells and in the lamina propria was expressed per 10,000 µm^2^. Values presented are means and SEM. Means annotated with different lower case letters above the bars are significantly different at *p* ≤ 0.05. For other explanations, see Figure 1.

**Figure 4:**
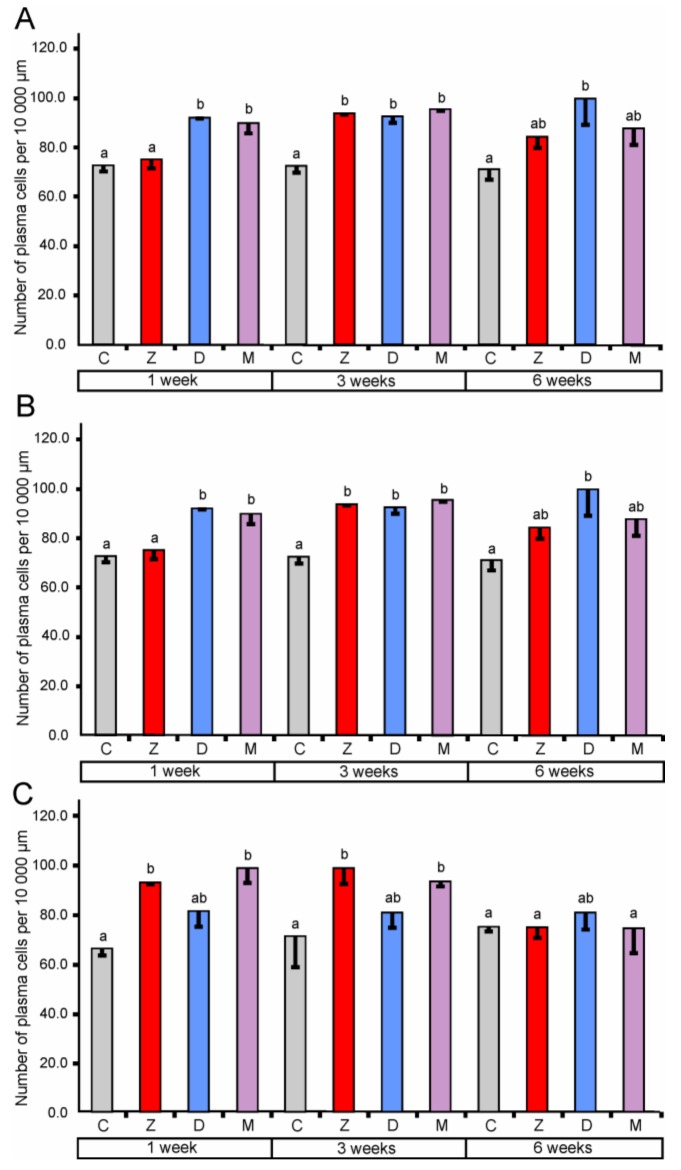
Number of plasma cells per 10,000 µm in the lamina propria of the cecum (**A**), the ascending colon (**B**), and the descending colon (**C**). Values presented are the means and SEM. Means annotated with different lower case letters above the bars are significantly different at *p* ≤ 0.05. For other explanations, see Figure 1.

In the cecum, quantitative analysis revealed a significant increase (compared to the corresponding control groups) in the number of lymphocytes in the epithelium after the treatment with ZEN for one, three, and six weeks. However, the administration of DON and ZEN + DON did not cause significant changes (see Figure 3). In the lamina propria of the cecum, significantly higher counts of lymphocytes were found after treatment with ZEN for one and three weeks, DON for one and six weeks, and ZEN + DON for three and six weeks. The plasma cell number was also influenced by mycotoxins in the cecum (see Figure 4). The significant increase in their number in the lamina propria was noted after treatment with ZEN for three weeks, with DON for one, three, and six weeks, and with ZEN + DON for one and three weeks (see Figure 4).

In the ascending colon, the relative number of lymphocytes in the mucosal epithelium showed a significant increase after three weeks of the treatment with ZEN and ZEN + DON (see Figure 3). Similarly, a significant increase in the number of lymphocytes was observed after ZEN and ZEN + DON treatment for three weeks in the lamina propria of the ascending colon (see Figure 3). Surprisingly, the number of plasma cells in the lamina propria of the ascending colon was lower than in the control pigs after three and six weeks of ZEN + DON administration (see Figure 4).

The administration of ZEN for three weeks and ZEN + DON for one week and three weeks resulted in a significant increase in the relative number of lymphocytes in the mucosal epithelium of the descending colon (see Figure 3). No significant changes were noted in the number of lymphocytes in the lamina propria of the descending colon (see Figure 3). In contrast, the number of plasma cells was significantly elevated in this part of the large intestine after one and three weeks of ZEN and ZEN + DON treatments (see Figure 4).

Based on the obtained results, it could be stated that ZEN stimulates the local immune system in the large intestine as indicated by the increase in the lymphocyte number in the mucosal epithelium as well as lymphocytes and plasma cells numbers in the lamina propria. The response of the intestinal immune system was unambiguous and clear in the cecum while more variable and sometimes difficult-to-interpret results were obtained in the ascending colon and the descending colon. It is a well-known phenomenon that the response of the immune system including the local subsystems to immunomodulators is dependent on several factors. Such variability was also reported in a case of mycotoxin effects on intestinal immune response in vivo and in vitro [37,38].

Mechanism of ZEN action on the intestinal immune system is poorly understood. ZEN in vivo and in vitro could activate the ROS-mediated NLRP3 inflammasome and, in turn, contribute to the caspase-1-dependent activation of the inflammatory cytokines Il-1β and IL-18 [39]. The direct effect of ZEN on lymphocytes by estrogen receptors presented in these cells was also postulated [16,40]. The studies concerning the effect of ZEN on the intestinal immune system were concentrated almost exclusively on the small intestine [18,41]. ZEN at the daily dose of 40 μg/kg BW caused increase in lymphocyte number in the villus epithelium of the jejunum after one week of treatment [18]. On the other hand, the toxin had no effect on lymphocytes in the villus epithelium after three and six weeks of the treatment. The same results were found in the lamina propria. In the duodenum, no effects of ZEN on lymphocytes in the epithelium covering the villi were observed. However, the toxin caused an increase in the lymphocyte number in the lamina propria [17]. The most recent histological study revealed inflammatory cell infiltration and tissue damage in the colon of mice fed by gavage with ZEN [39]. 

The results of quantitative analyses revealed that DON has no effect on the number of lymphocytes in the epithelium of the examined parts of the large intestine. However, the treatment with DON increased the number of lymphocytes and plasma cells in the lamina propria of the cecum and the ascending colon. This effect was dependent on the duration of treatment. Previous studies demonstrate the modulatory effects of DON on the mucosal immune response [3,42]. DON has been found to stimulate the production of mucosal antibodies [43] and IgA by Peyer’s patches lymphocytes [44]. The mechanism of the DON-induced increase in the lymphocyte number in the epithelium and the lamina propria as well as plasma cells in the lamina propria is likely related to the activation of cytokine synthesis by this mycotoxin [8,11,45]. In porcine jejunal explants, DON (10 μM) caused a significant increase in expression of mRNA encoding for Il-8, Il-1α, Il-1β, and TNF-α [11]. A phenomenon that DON induces an increase in the number of lymphocytes may also be related to the reduction of claudin and occludin production by this toxin, which enhanced permeability of the epithelial barrier [8,12]. In vitro, DON (0.5–1 µg/mL) contributed to the increase in permeability of *Salmonella Typhimurium* through the intestinal epithelium [46]. On the other hand, in our previous in vivo study, DON (12 µg/kg BW) did not disturb the intestinal barrier in the jejunum [18]. It was observed that DON causes changes in the basal membrane composition that facilitate the migration of lymphocytes from the lamina propria [47]. In some studies, the decrease of lymphocyte infiltration in the pig jejunum was found after DON administration (1.5–3 mg/kg feed) for four to five weeks. 

When the experimental pigs received DON + ZEN, different types of interactions were noted depending on the segment of the large intestine and the duration of treatment. In the cecum, they were antagonistic in the case of lymphocytes in the mucosal epithelium or less than additive in the case of lymphocytes and plasma cells in the lamina propria. In the ascending colon, they were antagonistic-lymphocytes in the mucosal epithelium and plasma cells in the lamina propria or less than additive—lymphocytes in the lamina propria. In the descending colon, they were additive or less than additive-lymphocytes in the mucosal epithelium and plasma cells in the lamina propria.

From previous studies on the small intestine it appears that a co-contaminated diet can cause several types of interactions. Bracarense et al. [12] investigated the effect of food contaminated with low doses of DON + FB (fumonisin) on the small intestine (jejunum and ileum) and stated synergistic (immune cells), additive (cytokines and junction protein expression), less than additive (histological lesions and cytokine expression), antagonistic (immune cells and cytokine expression) interactions of these mycotoxins. The authors’ [12] data provide strong evidence that chronic ingestion of low doses of mycotoxins alters the intestine and, therefore, may predispose animals to infections by enteric pathogens. Our results concerning the jejunum showed that the effect of ZEN and DON administered in combination is antagonistic on plasma cells after one, three, and six weeks of treatment, and on lymphocytes in the villus epithelium and the lamina propria after one week of treatment as well as less than additive on lymphocytes in the villus epithelium and in the lamina propria after three and six weeks of treatment [18]. In the duodenum, distinct effects were noted including additive on lymphocytes in the villus epithelium after one week of treatment, less than additive on lymphocytes in the epithelium and the lamina propria after three and six weeks, and on plasma cells in the lamina propria after six weeks, and antagonistic on lymphocytes (after one week) and plasma cells (after one and three weeks) in the lamina propria [17].

#### 2.1.4. Expression of the Proliferating Cell Nuclear Antigen (PCNA) in the Mucosa Epithelium

**Figure 5:**
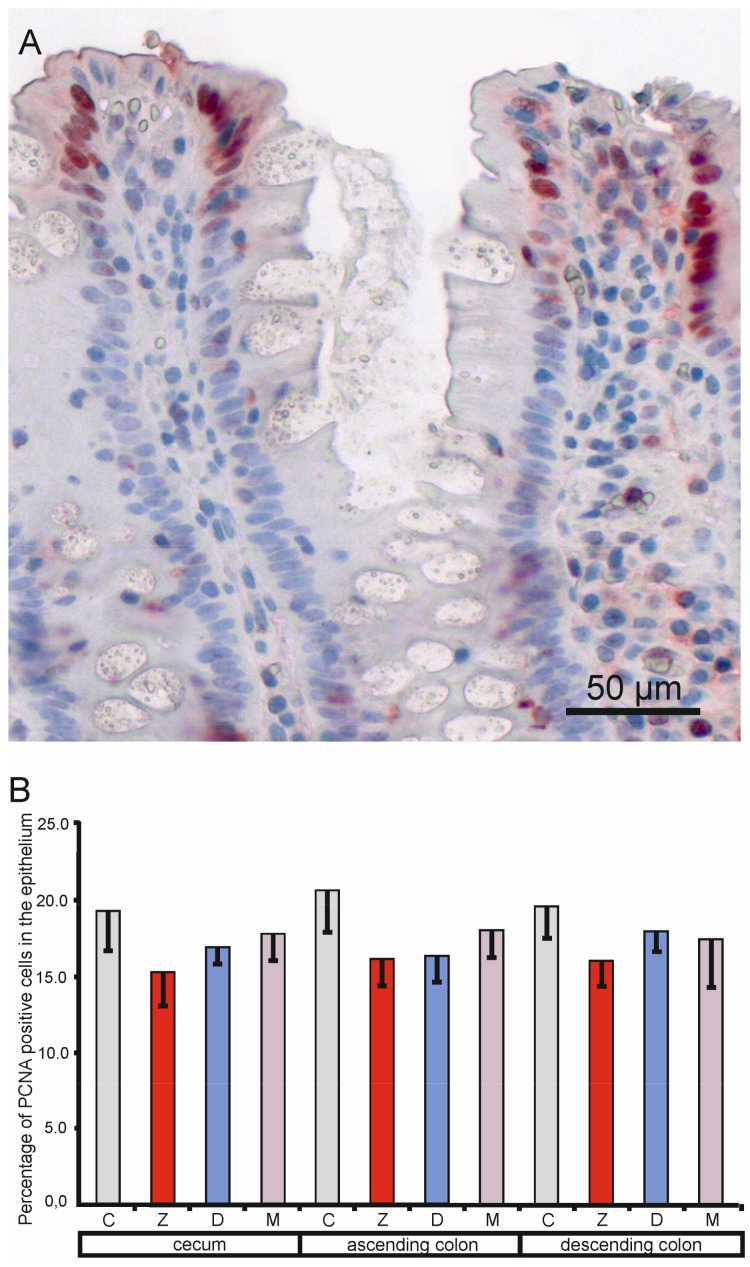
Effects of toxins on the proliferating cell nuclear antigen labeling index in the mucosal epithelium of the cecum, ascending colon, and descending colon in pigs after six weeks of treatment. (**A**) Immunopositive cells. (**B**) Percentage of proliferating cell nuclear antigen (PCNA) positive cells in the cecum, ascending colon, and descending colon. Values presented are means and SEM. For other explanations, see Figure 1.

PCNA positive cells were observed in the mucosal epithelium and in the lamina propria in all samples (see Figure 5A). The quantitative study performed on the samples taken at the end of the experiment did not show significant differences in the percentage of PCNA-positive cells in the mucosal epithelium between the control pigs and the animals treated with mycotoxins (see Figure 5B). 

The PCNA index is generally considered a valid measure of cell proliferation. Mycotoxins show an anti-proliferative effect on different cells. A significant decrease in expression of the proliferation marker PCNA was noted as effects of beta-zearalenol and DON on porcine endometrial cells in vitro [48]. DON in vitro inhibited the proliferation of human intestinal Caco-2 cells in a dose dependent manner with a significant effect appearing at 0.2 µg/mL [49]. The PCNA indexes for the jejunum and ileum in the piglets receiving DON (basal diet + 4 mg/kg of toxin) were significantly lower (*p* < 0.05) than those in the piglets that received the basal diet only [50]. In the present study, no negative effects of DON and ZEN on the PCNA labeling index were observed.

### 2.2. Electron Microscopy Study

#### 2.2.1. Ultrastructure of the Mucosa

In the control pigs, the mucosal epithelium of the cecum, ascending colon, and descending colon comprised mainly absorptive cells and goblet cells. Enteroendocrine cells and regenerative cells were less frequently observed. Lymphocytes were present between epithelial cells. The absorptive cells were columnar with a basally situated, oval nucleus. Their cytoplasm contained numerous ribosomes, profiles of rough and smooth endoplasmic reticulum, mitochondria, the moderately-developed Golgi apparatus, and a network of microtubules and filaments. Some of these cells comprised small, round granules with a content of variable electron density located in the upper part of the cell. The absorptive cells formed microvilli on the apical surface, whose number varied from cell to cell. The goblet cells were more or less elongated with a cup-shaped upper part, which was filled with large granules showing moderate electron density. The tall, lower part of these cells contained a cell nucleus, numerous cisterns of rough endoplasmic reticulum, the well-developed Golgi apparatus, mitochondria, and some secretory granules. The absorptive cells and goblet cells created junctional complexes in their upper parts. The intercellular spaces were frequently dilated beneath the junctional complexes and cells formed numerous irregular processes. Usually, the epithelium was covered with a layer of mucous. The lamina propria and the muscularis mucosae showed typical organization. Abundant lymphocytes, plasma cells, and macrophages were observed in the lamina propria. 

**Figure 6:**
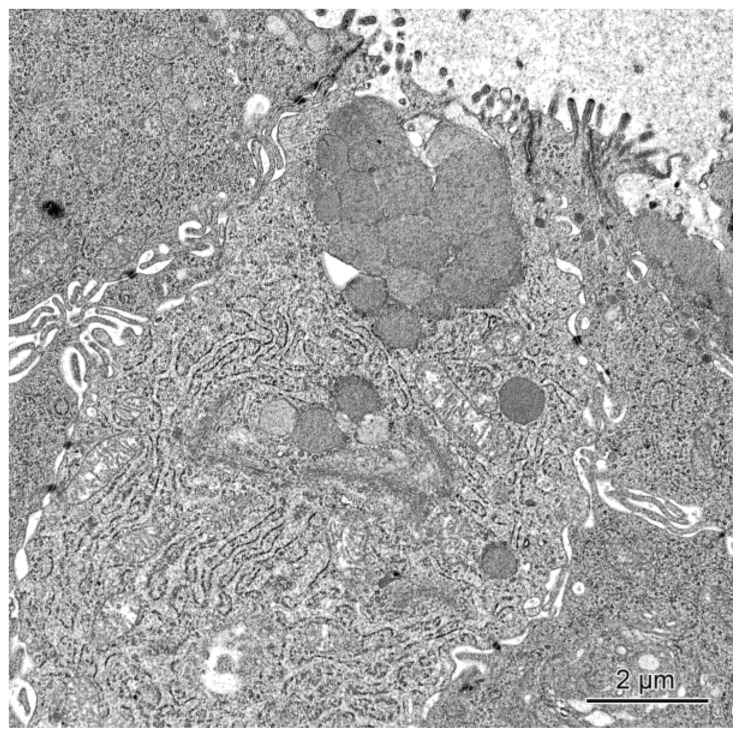
Goblet cell in the cecum of a pig receiving ZEN + DON for six weeks. Note the presence of sparse secretory granules in the apical part of the cell.

In the pigs treated with mycotoxins, the ultrastructure of mucosal epithelium showed some differences in comparison with the control animals. The most prominent changes concerned the goblet cell in the cecum and the descending colon of pigs receiving ZEN and ZEN + DON for one, three, or six weeks. In these pigs when compared to the control pigs, the goblet cells frequently contained much fewer granules in their apical parts (see Figure 6) and did not show a goblet-like shape. Our ultrastructural studies were only qualitative, however, it should be noted that the goblet cells were less frequently found in the group of pigs treated with ZEN and ZEN + DON than in two other investigated groups. 

**Figure 7:**
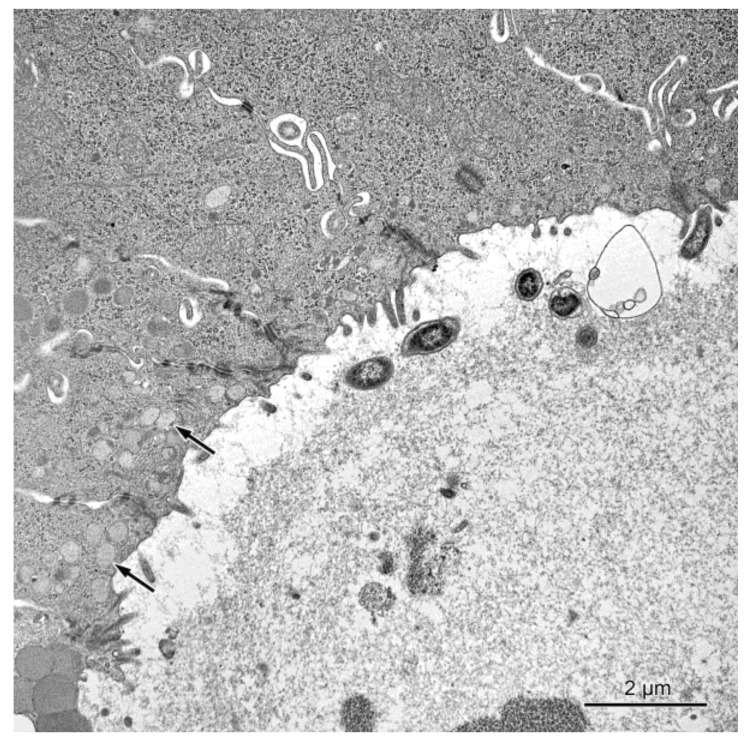
The transverse section through the intestinal crypt in the descending colon in a pig treated with ZEN + DON for three weeks. The apical surfaces of absorptive cells contain only single microvilli. Note the presence of numerous round granules in some absorptive cells (arrows).

**Figure 8:**
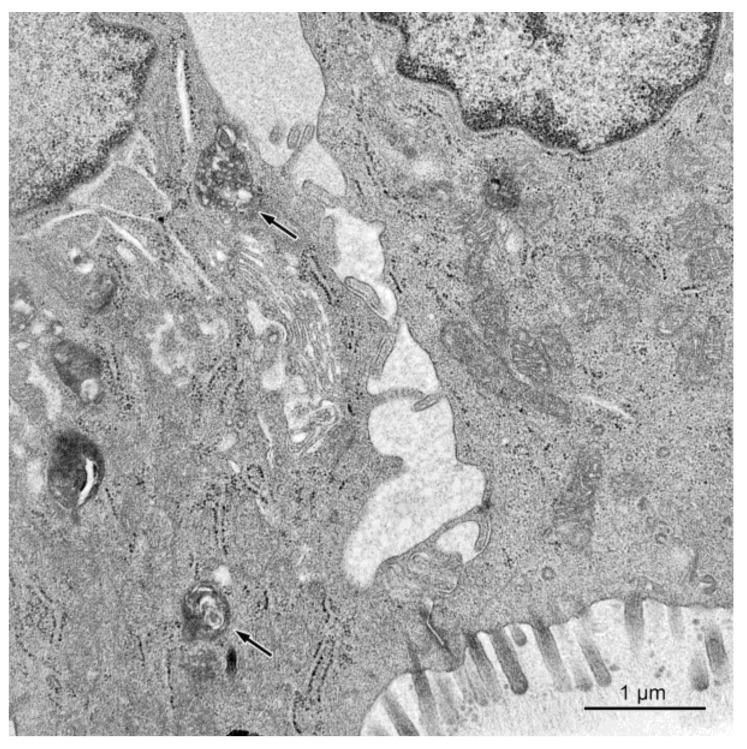
Dilated intercellular spaces between the epithelial cells in a pig treated with ZEN + DON for six weeks (ascending colon). Note presence of electron dense bodies in cytoplasm of the absorptive cell (arrows).

The alternations were also observed in the absorptive cells in the pigs treated with ZEN and ZEN + DON for one, three, and six weeks. As in the case of the goblet cells, they were most prominent in the cecum and descending colon. The apical surface of the absorptive cells of these pigs frequently formed only sparse microvilli, which usually were short and irregular (see Figure 7). Moreover, numerous small granules with moderate to high electron density were found in the upper parts of many absorptive cells (see Figure 7). In the group receiving ZEN + DON, some absorptive cells contained numerous electron dense bodies (see Figure 8).

The intercellular spaces between the epithelial cells were frequently largely dilated in pigs treated with DON and ZEN + DON for three and six weeks in the ascending and descending colon (see Figure 7). The damaged or dead epithelial cells were noted in all studied groups, but they were more frequently found in pigs treated with ZEN + DON for three and six weeks. The lamina propria of the large intestine in the animals treated with ZEN + DON for three and six weeks contained numerous macrophages, which were much less frequently observed in other groups of pigs.

Summing up, the results obtained in ultrastructural studies showed that ZEN and ZEN + DON affected mainly goblet cells, which often contained fewer secretory granules than in the control animals. Further studies are needed to determine if the synthesis of granules is decreased or the release of their content is increased. Both toxins also induced some changes in absorptive cells.

The literature data about effects of mycotoxins on the intestine ultrastructure are sparse and concerned almost exclusively with the small intestine. The administration of ZEN, DON, and ZEN + DON in the same dosages and according to the same schedule as in the present study did not cause changes in the ultrastructure of the mucosa of the jejunum with exception for adsorptive cells with drop-like protrusions of apical cytoplasm in the pigs receiving ZEN and DON + ZEN [17]. The treatment of gilts with ZEN at doses of 200 and 400 μg/kg BW for seven days had no effect on the ultrastructure of the jejunal epithelium [10]. In in vitro studies, incubation of jejunal explants with 10 μM DON resulted in an increase in intercellular spaces, a decrease in the size and number of microvilli, and a loss of junction complexes [51].

#### 2.2.2. Permeability of the Intestinal Barrier: Studies Using Lanthanum Ions

**Figure 9:**
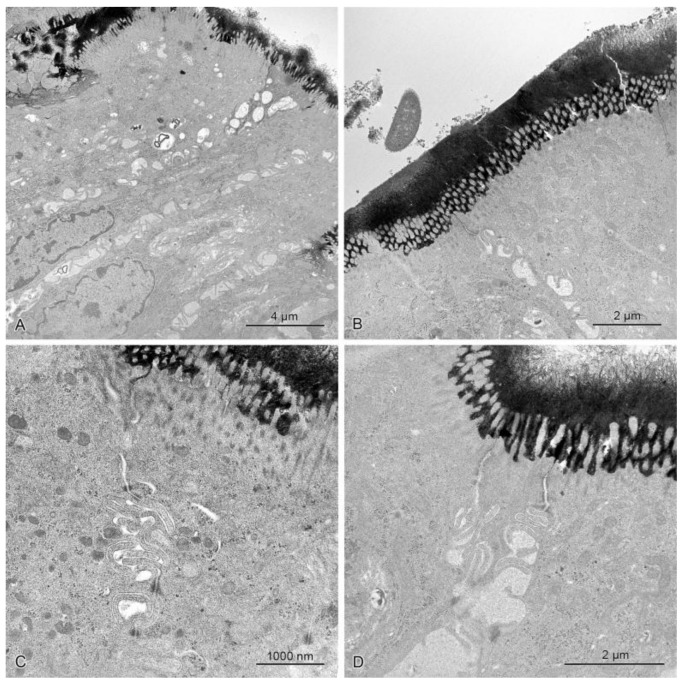
The mucosal epithelium in the samples of the descending colon in control pigs (**A**) and pigs treated with ZEN (**B**), DON (**C**), ZEN + DON (**D**) for six weeks. Note numerous precipitates on the epithelium surface and their lack beneath the tight junctions. The intercellular spaces did not contain any precipitates.

For evaluation of the effect of studied mycotoxins on the permeability of the epithelial barrier, we applied the lanthanum technique, which is commonly used in examining tight junctions at the ultrastructural level [52,53]. In the investigated material, electron dense lanthanum particles were observed on the surface of intestine epithelium between the microvilli and in intercellular spaces higher up in the tight junctions. The presence of deposits was never observed in intercellular spaces beneath the tight junctions (see Figure 9). 

The obtained results demonstrated that the treatment of DON, ZEN, and ZEN + DON did not change the permeability of epithelium in the examined segments of the large intestine. The lack of junction complexes on enterocytes exposed to mycotoxins was reported by Basso et al. [51]. Pinton et al. [54] associated alterations of barrier function in the pig jejunum after treatment with DON with a specific reduction in the expression of claudin. It should be stressed that the doses of toxins used in our study had no effects on the epithelial barrier in the jejunum [18]. 

## 3. Experimental Section

### 3.1. Toxins, Animals, and Experimental Design

Deoxynivalenol and zearalenon were synthesized and standardized at the Department of Chemistry, Faculty of Wood Technology, Poznań University of Life Sciences, Poland. Assessment of toxin purity was made using mass spectrometry (UPLC/TQD, Waters, Milford, MA, USA). The analytical purity of ZEN and DON were higher than 99.9%.

The study was performed on 36 clinically healthy gilts of mixed breed (White Polish Big × Polish White Earhanging) with body weights of 25 ± 2 kg at the beginning of the experiment. The animals were purchased from a farm where they received feed without detectable amounts of ZEN, DON, α-zearalenol, aflatoxin, and ochratoxin. The serological test excluded the presence of Auyeski’s disease, mycoplasmosis, parvovirosis, actinobacillosis, and porcine reproductive-respiratory syndrome. The pigs were fed twice daily and had free access to water. The tests for the presence of mycotoxins in feed were performed as described previously [55]. 

The animals were divided into four experimental groups (Z, D, and M; *n* = 9 in each group) and a control group (C; *n* = 9). The animals of the group Z received ZEN at a dose of 40 μg/kg BW per day, the animals of the group D—DON at a dose of 12 μg/kg BW per day, and the animals of group M—a mixture of ZEN and DON (40 μg ZEN/kg BW + 12 μg DON/kg BW per day). The mycotoxins were administrated per os during the morning feeding in water-soluble capsules containing oat bran as a vehicle. The gilts were weighed every week to establish the amount of DON and ZEN given for each animal. The animals of group C received capsules without mycotoxins.

Three animals from each experimental group were killed by intravenous (marginal vein of the ear) administration of sodium pentobarbital (Vetbutal, Biowet, Poland) at a dose of 140–150 mg/kg and exsanguination after 1, 3, and 6 weeks of the experiment. The tissue samples were taken 3 min after cardiac arrest.

All procedures were carried out in compliance with Polish legal regulations, which determine the terms and methods for performing experiments on animals, and the European Community Directive for the ethical use of experimental animals. The protocol was approved by the Local Ethical Council in Olsztyn (opinion No. 88/N of 16 December 2009).

### 3.2. Histological Examinations

The tissue samples (approximately 1 × 0.5 cm) were cut from the middle parts of the cecum and the ascending and descending colon. They were flushed in saline and fixed in 4% paraformaldehyde in 0.1 M phosphate buffer (pH 7.4) for 48 h, dehydrated in ethanol (TP 1020, Leica, Wetzlar, Germany), and embedded in paraffin (EG1150, Leica, Wetzlar, Germany). The 4-µm-thick sections were prepared with the use of HM 340E microtome (Microm, Lugo, Spain) and stained with the hematoxylin and eosin method (HE), periodic acid Schiff method (PAS), and methyl green-pyronine method (MGP) using automated multistainer ST 5020 (Leica, Wetzlar, Germany). The slides were signed in a way that prevented the people involved in their microscopic analysis from knowing the kind and duration of the animal treatment. The specimens were analyzed and photographed in an Axioimager light microscope equipped with an AxioCam MRc5 camera (Carl Zeiss, Oberkochen, Germany).

For morphometrical evaluations, the sections were scanned in a Mirax Desk scanner (Carl Zeiss, Oberkochen, Germany). The following parameters were determined: the thickness of mucosa and submucosa, the percentage of goblet cells in the surface epithelium covering the mucosa, the number of lymphocytes per 50 epithelial cells, the number of lymphocytes, and the number of plasma cells per 10,000 µm^2^ of the lamina propria. Goblet cells were counted in PAS-stained sections and plasma cells in MPG stained sections. All other measurements were performed on HE-stained sections. The linear measurements were repeated 20 times per animal. The percentage of lymphocytes and goblet cells were determined by counting their number per 50 epithelial cells in 10 randomly selected areas and the density of lymphocytes and plasma cells in the lamina propria were measured by counting the cells in 10 randomly selected areas of 6000 to 12,000 µm^2^ each. The measurements were performed using Pannoramic Viewer software (Version 1.15.3 RTM, 3D-Histech, Budapest, Hungary, 2012) and AxioVision software (Version 4.6.3, Carl Zeiss, Oberkochen, Germany, 2007). 

### 3.3. Immunohistochemical Staining

The staining was performed on paraffin sections mounted on Superfrost Plus slides prepared from the tissue samples taken for histological examinations after 6 weeks of the treatment. After dewaxing in xylene and rehydration in graded alcohols (96%, 70%, 50%), tissue sections were subjected to heat antigen retrieval in a microwave oven (600 W, 15 min) in 0.01 mol·L^−1^ 0.03% H_2_O_2_. Then slices were washed in phosphate buffered saline (PBS) and incubated for 20 min with 10% normal goat serum. The sections were then incubated overnight with primary antibodies anti-PCNA (clone PC10, M0879, Dako Agilent Technologies, Santa Clara, CA, USA) at a dilution 1:200 in a humidified chamber. Next, they were incubated with secondary antibodies for 30 min at a room temperature (EnVision System-HRP/AEC, Dako K4004, Santa Clara, CA, USA). For visualization of antigen-antibody complexes, the sections were immersed in 3-amino-9-ethyl-carbazole substrate-chromogenic (EnVision System-HRP/AEC, Dako K4004, Santa Clara, CA, USA) for 20 min. Afterwards, they were counterstained with Mayer’s hematoxyline (Sigma-Aldrich, St. Louis, MO, USA) and mounted using Mounting Glycergel (C0563, Dako Agilent Technologies, Santa Clara, CA, USA). The specimens were analyzed and photographed in a motorized Axioimager light microscope equipped with an AxioCamMRc5 camera (Carl Zeiss, Oberkochen, Germany). The PCNA labeling index (LI) was expressed as the ratio of cell positively stained for PCNA to all epithelial cells in at least 5 areas randomly selected for counting. The positive cell nuclei were counted on the images with AxioVision software (Version 4.6.3, Carl Zeiss, Oberkochen, Germany, 2007). 

### 3.4. Ultrastructural Examinations

The samples of the mucous membrane from the cecum, ascending colon, and descending colon were collected from sites adjacent to the sites of sampling for histological examination. The tissues were immersion-fixed in a mixture of 1% paraformaldehyde and 2.5% glutaraldehyde in 0.2 M phosphate buffer (pH 7.4) for 2 h at 4 °C, washed, and post-fixed in 2% osmium tetroxide for 2 h. After dehydration, the samples were embedded in Epon 812. Semithin sections were cut from each block of tissue, stained with 1% toluidine blue, and examined under a light microscope in order to choose the sites for preparing ultrathin sections. Ultrathin sections contrasted with uranyl acetate and lead citrate were examined with a Tecnai 12 Spirit G2 BioTwin transmission electron microscope (FEI, Hillsboro, OR, USA) equipped with two digital cameras: Veleta (Olympus, Tokyo, Japan) and Eage 4k (FEI, Hillsboro, OR, USA).

### 3.5. Lanthanum Procedure

The mucosal specimens were fixed for 2 h in a freshly prepared mixture of 2.5% glutaraldehyde and 1% lanthanum nitrate, La(NO_3_)·6H_2_O, in cacodylate buffer (pH 7.2). Next, the samples were rinsed for 30 min in the cacodylate buffer containing 1% lanthanum nitrate and postfixed for 2 h in a freshly prepared mixture of 1% osmium tetroxide and 1% of lanthanum nitrate in the cacodylate buffer. Then, the tissues were dehydrated and embedded in Epon 812. Unstained, ultrathin sections were examined with a Tecnai 12 Spirit G2 BioTwin transmission electron microscope (FEI, Hillsboro, OR, USA).

### 3.6. Statistical Analysis

The data were analyzed using two-way or one-way ANOVA with the Duncan test as a post-hoc procedure. Statistical analyses were performed by using Statistica software (Version 10.0 PL, StatSoft, Tulsa, OK, USA, 2011).

## 4. Conclusions

Taken together, the obtained data provide evidence that administration of low doses of DON and ZEN as well as a mixture of both toxins does not affect the architecture of the mucosa and submucosa in the large intestine of the pig. However, the treatment with toxins alters the system of goblet cells and the amount of lymphocytes and plasma cells in the mucosa. Modifications caused by mycotoxins vary depending on the duration of intoxication, the toxin, and the part of the large intestine. ZEN and ZEN + DEN significantly decrease the number and modify the ultrastructure of goblet cells. Taking into consideration the position of goblet cells in the mucosal biology especially in the mucosal protective mechanisms, the observed changes should be considered unfavorable for large intestine homeostasis. The results concerning lymphocytes and plasma cells are not unequivocal. However, they point to the influence of toxins on immune processes in the large intestine and pay attention to their complexity. Examinations of the PCNA labeling index and permeability of the intestinal epithelium show no significant effects of the examined mycotoxins on these parameters. The obtained results indicate that mycotoxins especially ZEN may influence the defense mechanisms of the large intestine.
